# Non-diffracting multi-﻿electron vortex beams balancing their electron–electron interactions

**DOI:** 10.1038/s41467-017-00651-z

**Published:** 2017-09-21

**Authors:** Maor Mutzafi, Ido Kaminer, Gal Harari, Mordechai Segev

**Affiliations:** 1 Physics Department and Solid State Institute, Technion, Haifa 32000 Israel; 2 Department of Physics, Massachusetts Institute of Technology, 77 Massachusetts Avenue, Cambridge, MA 02139 USA

## Abstract

The wave-like nature of electrons has been known for almost a century, but only in recent years has the ability to shape the wavefunction of EBeams (Electron-Beams) become experimentally accessible. Various EBeam wavefunctions have been demonstrated, such as vortex, self-accelerating, Bessel EBeams etc. However, none has attempted to manipulate multi-electron beams, because the repulsion between electrons rapidly alters the beam shape. Here, we show how interference effects of the quantum wavefunction describing multiple electrons can be used to exactly balance both the repulsion and diffraction-broadening. We propose non-diffracting wavepackets of multiple electrons, which can also carry orbital angular momentum. Such wavefunction shaping facilitates the use of multi-electron beams in electron microscopy with higher current without compromising on spatial resolution. Simulating the quantum evolution in three-dimensions and time, we show that imprinting such wavefunctions on electron pulses leads to shape-preserving multi-electrons ultrashort pulses. Our scheme applies to any beams of charged particles, such as protons and ion beams.

## Abstract (web summary)

Vortex electron beams are generated using single electrons but their low beam-density is a limitation in electron microscopy. Here the authors propose a scheme for the realization of non-diffracting electron beams by shaping wavepackets of multiple electrons and including electron–electron interactions.

## Introduction

The wave-like nature of electrons is now a well-established concept for many years, with famous experimental demonstrations such as the double slit experiment^1^ and Snell’s like refraction^2^. The wavelength of an electron with accessible energy is several orders of magnitude shorter than optical wavelengths, thereby providing access to microscopy experiments at atomic resolution^3^. Naturally, electron beam sources find numerous applications beyond microscopy, including radiation sources such as free electron lasers^4, 5^, electron beam lithography etc. Yet only in the past several years, the actual shaping of the wavefunction of electrons has become experimentally possible^6^. Indeed, shaping and manipulating the wavefunction of an electron is currently achieved through new techniques that use binary masks^7^ (made from a thin metal foil fabricated at nanoscale resolution) or amplitude and phase masks^8^ (made of thin silicon-nitride (SiN) membranes) imprinting the actual amplitude and phase distribution of the wavefunction. With these techniques, shaping the quantum wavefunction of electron beams (EBeams) has been used to generate EBeams carrying orbital angular momentum (OAM)^6, 7, 9, 10^ self-accelerating (Airy) EBeams^11^ and more recently—Bessel EBeams^12^. The ability to create vortex EBeams (EBeams that carry OAM) has opened a whole field of research and many studies has investigated and showed the properties and the features of vortex EBeams^6–10, 12–17^. Such techniques may also fundamentally change all EBeam applications and experiments, since they allow direct control over the quantum wavefunction of electrons^13^.

One of the most important applications of EBeams is electron microscopy, which has become an essential tool in many fields of science and technology, such as biology, materials science, electrical engineering and more. Scanning electron microscopy^18^ (SEM), transmission electron microscopy^19^ (TEM) and scanning transmission electron microscopy (STEM) produce images by scanning a sample with a focused EBeam or transmitting an EBeam through the sample. The EBeam interacts with the sample and produces an image containing information that is often at atomic resolution.

Importantly, the fundamental limit on the highest resolution possible in electron microscopy is the wavelength of the particle, which for electrons is on the order of picometers (10^−12^ m). In practice, however, state-of-the-art electron microscopes are still 2–3 orders of magnitude away from this fundamental limit, in spite of the recent advances in correcting aberrations. There are several reasons limiting the resolution of electron microscopes^20, 21^, among them the interaction between electrons^22, 23^, which is called the space-charge effect. This effect is currently the dominant resolution barrier in time-resolved electron microscopy^22–26^. Moreover, with the recent technological improvements in electron microscopy, the space charge effect is likely to remain the last fundamental issue constraining the resolution limit. Of course, when the density of the electrons in the beam is low enough, this effect becomes negligible. However, working with one electron at a time^23^ implies longer integration times in the detection process to obtain a reasonable signal-to-noise ratio (SNR). This space charge effect is of even greater importance in low-voltage electron microscopes, which are now becoming more popular. There, electron–electron interaction is already preventing an even lower acceleration voltages^27^. Moreover, in the past few years, novel experiments employ ultrashort pulses of electrons in microscopy, triggered by ultrashort pulses of light. These ultrashort pulses of electrons correspond to high electron density, which fundamentally limits the resolution due to space charge field effects^24, 25, 28^.

In principle, shaping the quantum wavefunction of electrons has the potential to improve the performance of traditional electron microscopes. However, thus far most electron microscopes have been using only low electron currents, working with one electron at a time, where the space-charge effect is negligible. The intrinsic reason for that is that electron–electron interactions cause repulsion between the electrons, which broadens the EBeam, and fundamentally hampers the resolution. For this reason, most electron microscopes rely on relatively low currents, which can be fully described by single electrons.

Here, we develop a quantum wave shaping technique to compensate for the repulsion between electrons and generate a tightly focused high-density EBeam, which maintains its shape and width for large distances. We propose to do that by proper shaping of the quantum wavefunction of multiple electrons, so as to counteract both the repulsion and the diffraction-broadening. To find such a non-diffracting multi-electron beam, we formulate the multiple-electron Schrödinger equation, which is nonlinear due to the interaction among electrons. Then, we solve for the wavefunction that preserves its shape in time. Finally, we demonstrate, in a three-dimensional (3D)+time simulation, that our specifically designed multi-electron beam is non-diffracting also under ultrashort pulse operation, and is robust to the broad spectrum of a 200 fs pulse. Our technique facilitates the use of EBeams made up of multiple electrons without compromising on the spatial resolution. It enables higher SNR with short integration time, by working with high-density EBeams while exhibiting spatial resolution equal to the resolution of a single-electron. As such, it can contribute to all electron beams applications and experiments, such as electron microscopy, free electron lasers, electron beam lithography, accelerators etc. In addition, future studies can lead to fabrication of masks for heavier charged particles, such as beams of ions, protons and even muons. Our scheme still applies in such cases and gives significantly better results because heavier charged particles have much lower velocity for the same acceleration field. Moreover, it should be possible to apply our scheme to other problems causing resolution degradation of electron beams, for example, the high charge density at the source (the tip), as we suggest below.

## Results

### Derivation of the equations of motion

The multiple-electron Schrödinger equation contains terms of interaction between electrons. Those terms change the Schrödinger equation to become a nonlinearly coupled set of wave equations. The full many-body problem is complex because the number of degrees of freedom is proportional to the number of electrons, which is computationally intractable (for a large number of electrons) with classical computers. Thus, we apply a mean-field approach (the Hartree approximation) to reduce the problem to a smaller number of nonlinearly coupled wave equations, which can still describe a large number of electrons. Mean-field approximations are commonly used for free-electrons, such as plasma^29, 30^, EBbeams^31–33^ and many other fermionic systems^34^. It is important however to note that stochastic effects, which are beyond the mean-field theory, have proven rather relevant for the space-charge broadening of ultrashort electron pulses in certain circumstances^31, 33^. From these coupled equations, we find the nonlinear non-diffracting EBeam wavefunctions that can also carry OAM. In other words, we find multi-electron vortex beams that preserve their shape. Generally, these solutions are not square-integrable, similar to the Bessel and Airy beams^11, 12^. Hence, generating them in a physical setting implies truncating their wavefunctions, which makes the beam non-diffracting only for a finite distance. Simulating the evolution (propagation dynamics in space and time) of these beams shows that the range within which our non-diffracting multi-electron beams remain with negligible diffraction broadening can be very large, and proves their robustness to noise and to deviations from non-ideal launch conditions. Finally, we compare the non-diffraction range of our non-diffracting wavefunctions with that of a Gaussian EBeam (which is roughly the wavefunction that naturally occurs in electron microscopes) and with a Bessel EBeam (the non-diffracting analogue for a single electron). In all these simulations, we observe substantially larger non-diffraction range of our multi-electron wavefunctions. This paves the way for using properly shaped multi-electron beams in electron microscopy as well as in a variety of other applications.

Thus far, wavefunction shaping of EBeams^7, 8, 10–12^ have only considered EBeams comprising of a single electron. As such, these methodologies are inapplicable for high-density electrons beams, where electronic repulsion is appreciable. At the same time, high-density EBeams are encountered in a variety of applications, ranging from accelerators, high-current and low-voltage electron microscopy (LEEM) to high-intensity X-ray sources (e.g., Free Electron Laser) and much more. Naturally, controlling and shaping high-density EBeams is also important from the basic science view point.

The repulsion between electrons renders the beam diffraction to be density-dependent, thereby making the problem nonlinear. That is, while a sufficiently dilute EBeam is described by the linear Schrödinger equation, the physics becomes considerably more complex when many-body interactions take place. This draws a fundamental difference between electron beams and electromagnetic beams: at intensities lower than 10^22^ Watts cm^−2^, where vacuum quantum electrodynamics effects are negligible^35^, photons do not directly interact with one another, whereas electrons inevitably always interact with one another.

Importantly, in addition to Coulomb repulsion, EBeams are also subject to spin–spin interaction, and in case of beams carrying OAM, also to spin–orbit interaction, thereby adding additional complexity to the dynamics^14^. However, under the typical parameters of electron microscopes (primarily that all features are much larger than the Compton wavelength), the spin–spin and spin–orbit interactions are negligible compared to the electrostatic potential energy (see Supplementary Note 3 for details).

The exact Schrödinger equation contains a nonlinear set of coupled equations whose number is the number of electrons. To make the problem tractable, we approximate the full multi-electrons Hamiltonian by the Hartree Hamiltonian, which is an effective mean-field Hamiltonian. This approach assumes that the influence of the fermionic nature of the electrons (the exclusion principle) is very weak^36, 37^ (see discussion in the Supplementary Note 1). Hence, we are allowed to take the simplest case where all the electrons have the same wavefunction, as happens naturally in electron microscopes. We also restrict the wavefunction to be cylindrically symmetric while allowing it to carry OAM. This wavefunction is therefore of the form

1 $$\psi \left( {{\bf{r}},t} \right) = \frac{1}{{{a_0}}}\phi \left( \rho \right){e^{il\theta }}\frac{{{e^{ikz - i\omega t}}}}{{\sqrt L }},$$

where, *a*_0_ is Bohr’s radius, *l* is the OAM and *k* is the wavenumber in the *z* direction. The normalization factor, $\sqrt L$, sets the characteristic length scale (*z*) within which the wavefunction is significant (the so-called uncertainty length). Although this factor cancels out later on, this length scale is useful for estimating the strength of the effects involved (see Supplementary Note 3). The time evolution of the wavefunction *ψ*, according to the Hartree Hamiltonian (see discussion in the Supplementary Note 1), is

2 $${- i\hbar {\partial _t}\psi \left( {{\bf{r}},t} \right) = - \frac{{{\hbar ^2}}}{{2m}}{\nabla ^2}\psi \left( {{\bf{r}},t} \right) + \frac{{N{e^2}}}{{4\pi {{\rm{\varepsilon }}_0}}}\left( {{\int} {\frac{{{{\left| {\psi \left( {{{\bf{r}}\prime },t} \right)} \right|}^2}}}{{\left| {{\bf{r}} - {{\bf{r}}\prime }} \right|}}{{\rm d}^3}{{\bf{r}}\prime }} } \right)\psi \left( {{\bf{r}},t} \right),}$$

where *ħ* is the reduced Planck constant, *m* and *e* are the mass and charge of the electron respectively, *ε*_0_ is the vacuum permeability and *N* is the total number of electrons in the EBeam. This equation is known as Choquard equation^38^. The second term in the right hand side of Eq. (2) resembles an effective potential. Hence, we define

3 $$U\left( {{\bf{r}},t} \right) = 2N{a_0}{\int} {\frac{{{{\left| {\psi \left( {{{\bf{r}}\prime },t} \right)} \right|}^2}}}{{\left| {{\bf{r}} - {{\bf{r}}\prime }} \right|}}{d^3}{\bf{r}}\prime} .$$

Substituting Eq. (3) in Eq. (2), we get the following coupled equations,

4 $$ - i\hbar {\partial _t}\psi \left( {{\bf{r}},t} \right) = \frac{{{\hbar ^2}}}{{2m}}\left( { - {\nabla ^2} + \frac{1}{{a_0^2}}U\left( {{\bf{r}},t} \right)} \right)\psi \left( {{\bf{r}},t} \right),$$

5 $${\nabla ^2}U\left( {{\bf{r}},t} \right) = - 8\pi N{a_0}{\left| {\psi \left( {{\bf{r}},t} \right)} \right|^2}.$$

Substituting the wavefunction from Eq. (1) as a source term in Eqs (4) and (5), we find that the effective potential also has rotational symmetry, and as such it depends only on *ρ*, such that *U*(**r**, *t*) = *U*(*ρ*). We now look for a solution that is non-diffracting, namely, we seek a wavefunction whose expectation value does not vary in time. This allows the separation into two coupled nonlinear differential equations,

6 $$ - \left( {\frac{1}{\rho }{\partial _\rho }\left( {\rho {\partial _\rho }} \right) - \frac{{{l^2}}}{{{\rho ^2}}}} \right)\phi \left( \rho \right) + \frac{1}{{a_0^2}}U\left( \rho \right)\phi \left( \rho \right) = 0,$$

7 $$\frac{1}{\rho }{\partial _\rho }\left( {\rho {\partial _\rho }} \right)U\left( \rho \right) = - \frac{{8\pi n}}{{{a_0}}}{\left| {\phi \left( \rho \right)} \right|^2},$$

where, *n* is the density of electrons per unit distance. These equations resemble the Newton–Schrödinger model^39, 40^, which is often used in General Relativity to describe the dynamics of wavefunctions under the gravitation potential they themselves induce. This set of equations also resemble the equations used to describe the dynamics of optical beams in the presence of the highly nonlocal optical thermal nonlinearity, which supports solitons^41^ and their long-range interactions^42^. Such an optical system was recently used to emulate effects predicted in General Relativity, and discover new phenomena^43^. Interestingly, this system also resembles the model describing long-range interactions between cold atomic dipoles^44^, which also give rise to solitons and related phenomena. These nonlocal solitons^41, 42^ and their counterparts in cold dipoles^44^ resemble the non-diffracting multi-electron wavepackets found here, as solutions to Eqs (6) and (7). However, whereas in the Newton–Schrödinger model the force is always attractive, the force here is always repulsive. This means that, while the nonlinear non-diffracting wavepackets in the Newton–Schrödinger model are solitons, and are therefore localized and square-integrable^41^, we expect the localized non-diffracting solutions of Eqs (6) and (7) to be not square-integrable. Intuitively, seeking localized solutions for Eqs (6) and (7) resembles searching for non-diffracting beams in self-defocusing thermal optical nonlinearities, which fundamentally cannot support bright solitons but can support dark solitons^45^ and also localized non-diffracting wavepackets that are not square integrable (e.g., nonlinear Bessel-like beams^46^).

### Finding the non-diffracting wavefunction

To solve these differential equations and find a shape-invariant solution, we need to determine the initial conditions. The wavefunctions we seek have rotational symmetry with respect to the propagation axis (*z*), hence so does the effective potential, which yields *U*′(*ρ* = 0) = 0. Substituting this condition into Eq. (6) at the vicinity of *ρ* = 0 gives the Bessel equation, whose solution is *ϕ*(*ρ*) ~ *αJ*_*l*_(*k*_*T*_ *ρ*) (the other solution, *Y*_*l*_(*k*_*T*_ *ρ*), is unphysical because it diverges at *ρ* = 0). Using *ϕ*(*ρ*) in Eq. (6) leads to $U\left( {\rho = 0} \right) = - k_T^2$, where *k*_*T*_ is a real positive number, which corresponds to the transverse momentum. Another initial condition is provided by the normalization requirement $2\pi {\int}_{\!{\rm{BSS}}} {{{\left| {\phi \left( \rho \right)} \right|}^2}\rho {\rm d}\rho = 1}$, where the integral boundaries correspond to the Beam Spot Size (BSS) defined at the plane where the beam is shaped. For a given BSS we get a continuous set of solutions, determined by the free parameter *k*_*T*_, which can vary between 0 and infinity.

In summary the initial conditions for Eqs. (6) and (7) are

8 $$\left\{ {\begin{array}{*{20}{c}} {\phi \left( 0 \right) = \alpha } \\ \\ {\phi '\left( \varepsilon \right) = \alpha {k_T}J_l^\prime \left( {{k_T}\varepsilon } \right)} \\ \\ {U\left( 0 \right) = - k_T^2} \\ \\ {U'\left( 0 \right) = 0} \\ \end{array}} \right.$$

with, normalization requirement,

9 $$2\pi {\int}_{\!\!\!\!{\rm{BSS}}} {{{\left| {\phi \left( \rho \right)} \right|}^2}\rho {\rm d}\rho = 1.}$$

We solve Eqs (6) and (7) with the initial conditions and the normalization condition from Eqs (8) and (9), numerically (see Supplementary Note 1), where *α* is found iteratively to fulfill requirement Eq. (9).

**Fig. 1:**
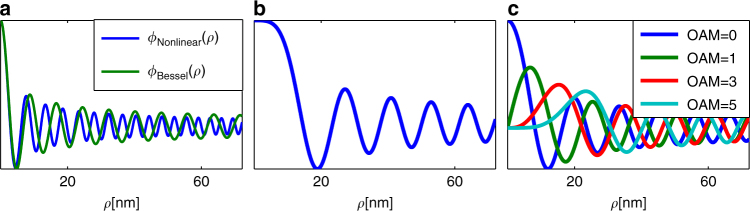
Radial part of the shape-invariant wavefunctions of multi-electron beams. **a** Radial wavefunction (*ϕ*(*ρ*)) of the beam with zero OAM (*blue*), compared with the Bessel function which is the corresponding non-diffracting single-electron beam. **b** The unique over-wide radial wavefunction obtained for *k*_*T*_ = 0, which does not allow OAM, and does not have a corresponding non-diffracting single-electron beam. **c** Radial wavefunctions of multi-electron beams carrying OAM = 0, 1, 3, 5 (*blue*, *green*, *red* and *cyan*, respectively)

Figure 1a shows an example of the radial wavefunction *ϕ*(*ρ*) of a non-diffracting EBeam with zero OAM. It is instructive to compare this wavefunction (which is a shape-invariant solution of the nonlinear equation) to the Bessel function, which is a shape-invariant solution of the linear equation describing the evolution of a single electron^12^. Figure 1a shows this comparison, with the same value of *k*_*T*_. In the vicinity of *ρ* = 0, the shape-invariant solution coincides with *J*_0_(*k*_*T*_ *ρ*), but for large *ρ* its lobes are considerably denser than those of the Bessel function. The reason is as follows. The Bessel beam is designed to compensate exactly for linear diffraction broadening, whereas our multi-electron beam displays additional broadening due to the nonlocal repulsion among electrons. Intuitively, to compensate for both the diffraction and the electron–electron repulsion, the lobes of the beam must be denser. That is, denser lobes carry more transverse momentum, which is required to compensate for the additional diffraction broadening caused by the multi-electron repulsion. Accordingly, the effective potential representing the repulsion between electrons, $U\left( \rho \right) = - k_T^2\left( \rho \right)$, is increasing with *ρ*, which implies that higher transverse momentum (denser oscillations) is required to compensate for the repulsion at higher *ρ* values. An interesting case is shown in Fig. 1b, which presents the radial function found for *k*_*T*_ = 0 (with *ϕ*′(0) = 0 and *ϕ*″(0) = 0), which yields the upper limit to the width of the main lobe of the radial wavefunction of the shape-invariant solutions of Eqs (6) and (7). This solution does not have a corresponding linear solution, because the Bessel function becomes a constant (corresponding to a plane wave) for *k*_*T*_ = 0. Figure 1c displays several radial wavefunctions of shape-invariant solutions that carry OAM (with the same value of *k*_*T*_ and BSS as in Fig. 1a). The blue, green, red and cyan curves correspond to OAM of zero, one, three and five, respectively.

### Creating the non-diffracting wavefunction

The non-diffracting wavefunctions (the solutions of Eqs (6) and (7)) can be generated by passing the EBeam through a binary holographic mask as in refs ^6, 7, 10–12^, or through a phase mask imprinting the actual phase distribution of the shape-invariant wavefunction^8^, which shapes the electron wavepacket directly. The holographic mask has the following transmission function

10 $${T_{{\rm{holographic}}\,{\rm{mask}}}} = {\left| {{\cal F}\left\{ {\phi \left( \rho \right){e^{il\theta }}} \right\} + {e^{i{k_h}\rho \,{\rm{cos}}\,\theta }}} \right|^2},$$

where *ϕ*(*ρ*)*e* ^*ilθ*^ is the non-diffracting wavefunction (solution of Eqs (6) and (7)), F is its Fourier transform, and ${e^{i{k_h}\rho \,{\rm{cos}}\,\theta }}$ is a plane wave acting as a reference for the hologram. This transmission function is designed to have a binary shape, as in refs ^6, 7, 10–12^ according to

11 $$T_{{\rm{holographic}}\,{\rm{mask}}}^{{\rm{binary}}} = \left\{ {\begin{array}{*{20}{c}}{1,} & {{T_{{\rm{holography}}}}\,  >\,{\rm{threshold}},\,{\rm{and}}\,\,\rho < {\rho _{{\rm{max}}}}} \\ \\ {0,} & {{\rm{else}}} \\ \end{array}} \right..$$

**Fig. 2:**
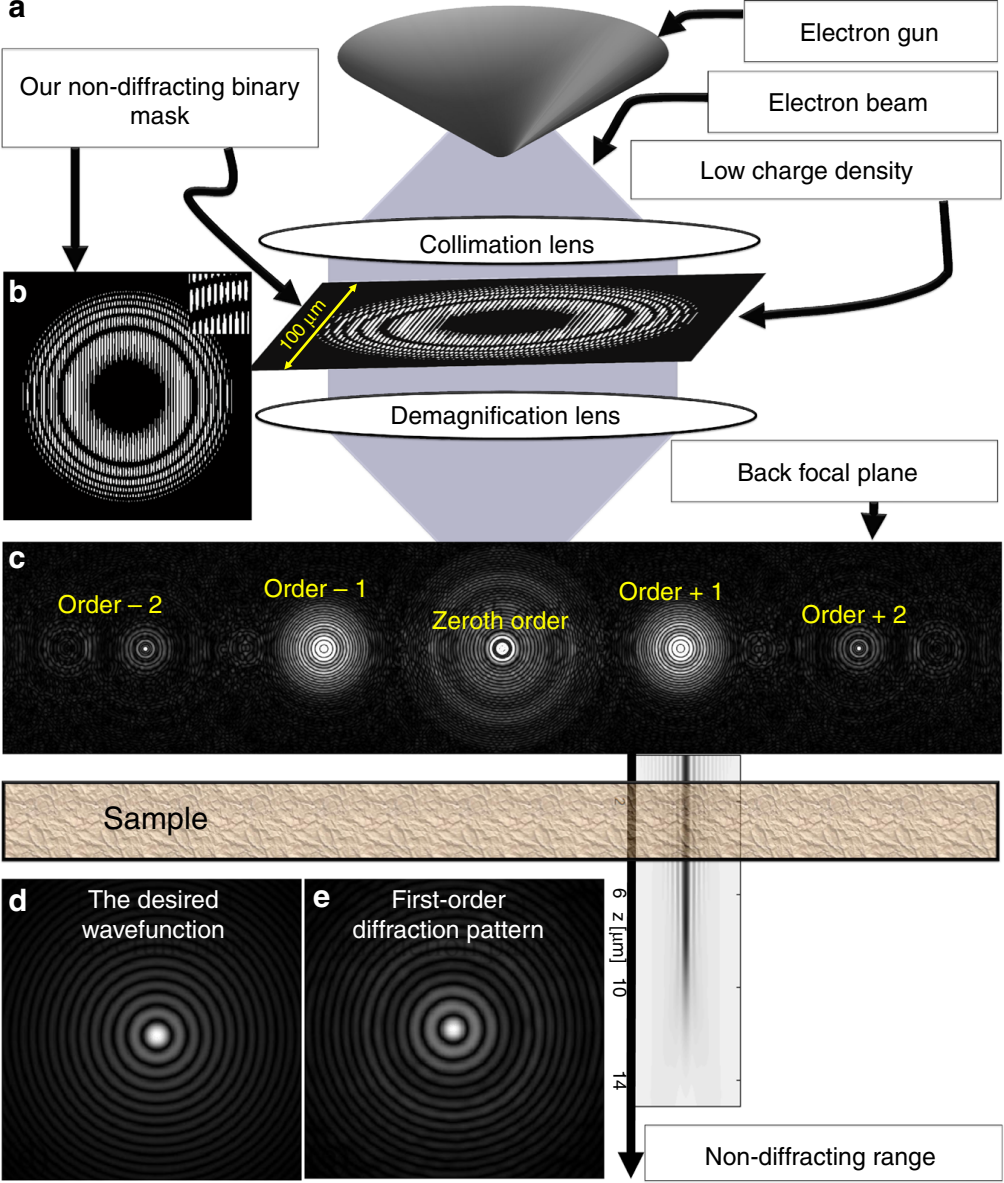
Holographic generation of the shape-invariant wavefunction of multi-electron beams. **a** An electron beam is transmitted through a binary with the shape from Eqs (10 and 11). Then it is focused by a magnetic lens to the Fourier domain at the back focal plane. **b** Transmission function of the binary mask. **c** The diffraction pattern generated by passing a plane wave through this mask at the back focal plane. The center pattern is the zeroth-order diffraction pattern. The pattern on the left (*right*) of the zeroth order corresponds to the +1 (−1) diffraction order. This diffraction pattern shows that the diffraction patterns can be cleanly separated from one another, and that the ±1 orders can be used to generate the non-diffracting wavefunction. **d**, **e** Comparison between the diffraction pattern obtained from the +1 order and the desired wavefunction for which the mask is designed. This demonstrates that a very good approximation of the desired wavefunction can be generated even with a binary mask fabricated with present technologies (of course adding a phase mask^8^ would give an even better result)

An example for an experimental scheme for generating the shape-invariant wavefunction of multi-electron beams is shown in Fig. 2. It is similar to the way Bessel EBeams^12^ and Airy EBeams^11^ are generated. Figure 2a shows the scheme, where the non-diffracting wavefunction multi-electron beam is generated by a holographic mask. An electron beam is transmitted through a binary mask, then focused by a magnetic lens to the back focal plane, which is where the sample is inserted. Importantly, the scheme ensures that the high density of electrons is obtained at the back focal plane, whereas the density at the mask plane is low (BSS = 10 μm), such that the electron–electron repulsion at the mask is weak. Thus, the beam experiences linear diffraction everywhere else in the system before the back focal plane. Another approach for generating the non-diffracting wavefunction of multi-electron beams may be to directly shape the tip of the electron gun (as done in refs ^47–51^), to the specific shape that generates our non-diffracting wavefunction. Doing this could also resolve the space-charge problem at the tip of the electron gun, which is currently where the space-charge effect is the most problematic in electron microscopy.

Figure 2b shows the transmission function of the binary holographic mask required for shaping the wavefunction shown in Fig. 1a. Figure 2c shows the diffraction pattern obtained at the back focal plane when passing a plane wave through this mask. The central waveform is the zeroth-order diffraction pattern. The non-diffracting wavefunction is on the left, and its complex conjugate appears on the right, corresponding to +1 and −1 diffraction orders, both having BSS of 140 nm. Figure 2d singles out the first diffraction order, showing a very good agreement with the desired pattern of Fig. 2e, which represents the absolute value of *ϕ*(*ρ*)—the non-diffracting wavefunction defined by Eqs (6) and (7).

### Evolution of multi-electrons beams

At this point it is important to simulate the propagation dynamics of the multi-electron beams we have found, which are meant to be shape-invariant for some finite propagation range, and compare them to the evolution of the Bessel beam (the diffractionless solution for a single-electron) and to the evolution of Gaussian EBeam. To facilitate a quantitative comparison, we truncate all three beams by the same BSS. The simulation method used for the 2D + 1 quantum system (described in Eqs (4) and (5)) is the beam propagation method (or split step Fourier method), with the addition of a procedure calculating the potential *U* at each step. Specifically, given an initial wavefunction of some shape *ψ*(*x*, *y*, *z*), we calculate the potential *U*(*x*, *y*, *z*) by solving numerically Eq. (5), for the initial conditions described in Eq. (8). Then, we use this potential in Eq. (4) to calculate the beam in the next step (*x*, *y*, *z* + d*z*), and so on (see Supplementary Note 2 for further details).

**Fig. 3:**
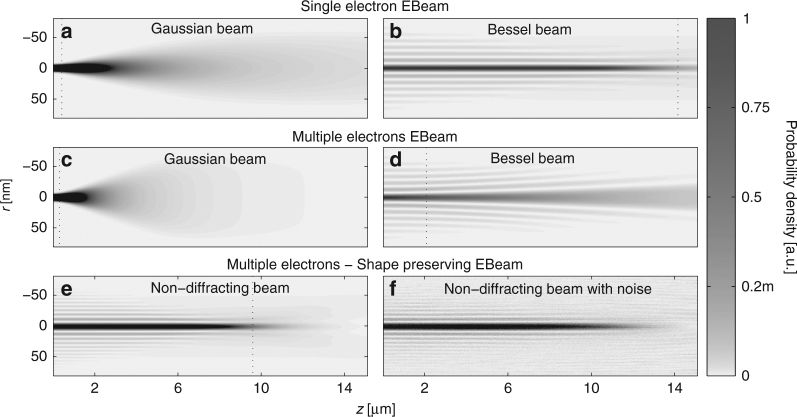
Evolution of the density of various single- and multi- electron beams. **a**, **b** Propagation of a single-electron beam (which does not have self-repulsion) with initial Gaussian and Bessel wavefunctions, respectively. **c**, **d** Propagation of the respective cases for EBeams containing multiple electrons. The repulsion among the electrons makes the Gaussian EBeam diffracts faster than in **a**, while the Bessel broadens considerably and is no longer shape preserving. **e** Propagation of the non-diffracting multi-electron beams with the wavefunction of Fig. 1a. The beam preserves its shape for a large distance in spite of the repulsion among electrons. **f** Same as **e** with added noise, exhibiting robustness to high level of noise (uniformly distributed Gaussian noise that carries the same power as the beam). The dashed lines in **a**–**e**, indicate the range of non-diffraction of each beam. Here, the EBeams are accelerated by voltage of 200 V, having beam current of *I* = 5 μA (for the multi-electron beams), and effective width of 16 nm (Eq. 12)

**Fig. 4:**
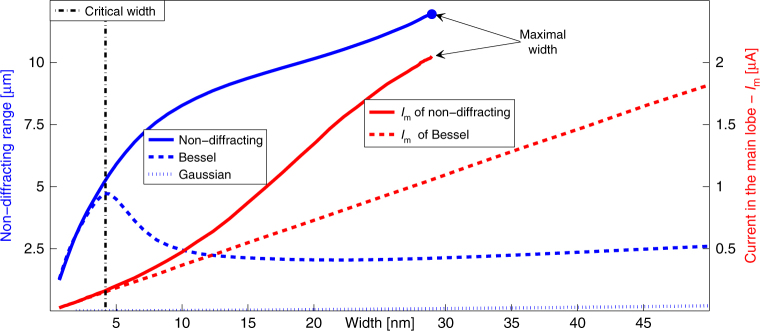
Non-diffraction range and effective current vs. beam width. The plot shows the non-diffraction range (*blue curves*) and effective current (*red curves*) vs. beam width, for our non-diffracting wavefunctions (*solid curves*), for multi-electron Bessel (*dashed curves*) and Gaussian (dotted curve) EBeams with zero OAM. The effective width of our non-diffracting multi-electron beam is bounded from above at the *blue dot*, where the main lobe is over-wide, carrying the upper limit on the current. This upper limit occurs because the interference of our shaped wavepacket can balance the beam self-repulsion and diffraction only up to a certain value, above which the beam spread is too strong for the predesigned interference effects (arising from the structure of the beam) to compensate for it. For beams of effective width much narrower than the critical width (marked by the dashed vertical line at 4.2 nm) our non-diffracting multi-electron wavefunction coincides with the Bessel function. This critical width decreases as the current is increased and can go below 1 nm. Here, the EBeams are accelerated by voltage of 200 V, having beam current of *I* = 5 μA

Figure 3 presents the simulated propagation dynamics of several wavefunctions, displaying the density of the EBeams as a function of *ρ* and the propagation distance *z*. In all of these examples, the acceleration voltage is 200 *V* (typical TEM energies) and the current is 5 μA, which is considered a very high current in microscopes, meant to highlight our findings (ref. ^36^ presents the use of such high current together with coherent tip and coherent EBeam). Here, it is important to note that an EBeam with acceleration voltage of 200 V and total current of 5 μA carries the same electron density (in the longitudinal direction, proportional to the ratio *I*/$\sqrt V$) as an EBeam with acceleration voltage of 20 kV and total current of 50 μA. Therefore, the simulations (Figs 3 and 4) correspond to both cases, up to a scaling constant in the propagation axis.

In coming to examine the diffraction-broadening effects during propagation of these beams, we recall that the resolution of electron microscopes is determined by the region of high density of electrons (high-current density). It is therefore natural to examine the width of the region of high electron density in the beam. Observing Fig. 3, we notice that our non-diffracting multi-electron wavefunctions exhibit diffraction effects that are fundamentally different than diffraction of Gaussian beams. Namely, whereas in Gaussian beams the width expands monotonically with distance, for the multi-electron non-diffracting wavefunctions—when the scheme (Fig. 2a) allows enough oscillating lobes, the main lobe maintains its width and shape for a very large distance. The finite extent of the non-diffracting wavefunctions is caused by the zeros around the main lobe filling up until the contrast between the main lobe and secondary lobes decreases and they merge and broaden. Before this occurs, the full-width at half-maximum (FWHM) of the main lobe of our non-diffracting multi-electron beam (Fig. 3e) varies very little (unlike the truncated Bessel beam whose FWHM varies considerably, as shown in Fig. 3d). Actually, the resolution of in electron microscopy is determined by the size of the region of high intensity (region of high probability). We therefore define a measure for effective width of the main lobe, as the second moment of the electron density, measured in the main lobe region (defined by the zero around it), as

12 $$w = \frac{{\sqrt {{\int} {{\int}_{{\rm{Main}}\,{\rm{lobe}}} {{{\left| {\psi \left( {x,y} \right)} \right|}^2}\left( {{x^2} + {y^2}} \right){\rm d}x{\rm d}y} } } }}{{\sqrt {{\int} {{\int}_{{\rm{Main}}\,{\rm{lobe}}} {{{\left| {\psi \left( {x,y} \right)} \right|}^2}{\rm d}x{\rm d}y} } } }}.$$

For the Gaussian wavefunction (which has no zeros), this effective width is simply the second moment. In all the examples in Fig. 3, the effective width is *w* = 16 nm. In this vein, we define the range of non-diffraction *L*_d_ as the distance for which the effective width is increased by a factor of $\sqrt 2$. The non-diffraction ranges of the various beams in Fig. 3 are marked by the horizontal dashed line in each panel.

Figure 3 shows the simulated evolution of several wavefunctions launched as initial conditions for solving Eqs (6) and (7), where the single-electron cases (Fig. 3a, b) do not include the effective potential term (*U*), while the multi-electron cases (Fig. 3c–f) include the nonlinear term representing the repulsion among electrons. Figure 3a, b present the propagation of single-electron beam of initial Gaussian and Bessel wavefunctions, respectively. Both wavefunctions are launched with BSS of 140 nm (at the back focal plane), which has no effect on the Gaussian beam (whose width is narrower than the 140 nm), but truncates the Bessel beam after 10 oscillatory lobes. The single-electron Gaussian beam exhibits fast diffraction (*L*_d_ = 420 nm), while the single-electron Bessel beam preserves its shape for a very large range distance (*L*_d_ = 14.16 μm). Figure 3c, d present the corresponding cases for EBeams comprising of multiple electrons. Clearly, the Gaussian multi-electron beam (Fig. 3c) expands faster (*L*_d_ = 300 nm) than the single-electron Gaussian beam (Fig. 3a), due to the repulsion among electrons. Likewise, the multi-electron Bessel beam (Fig. 3d) also expands faster (2.1 μm) compared to the single-electron case which ideally (had it not been truncated) would remain non-diffracting indefinitely (Fig. 3b). Clearly, the Bessel function is not a suitable non-diffracting solution of the nonlinear evolution (Eq. (2)). On this background, Fig. 3e presents the propagation dynamics of our non-diffracting wavefunction shown in Fig. 1a. Had this wavefunction not been truncated by the BSS, it would have preserved its shape indefinitely, in spite of the repulsion among electrons. The BSS truncates the wavefunction after 17 oscillatory lobes, and consequently causes the diffraction effects shown in Fig. 3e. Figure 3f shows the evolution of the same wavefunction in the presence of additive Gaussian noise. To highlight the robustness of our findings, Fig. 3f simulates the extreme case, where the total noise current is equal to the total current carried by the BSS beam of Fig. 3e, and is uniformly distributed in real-space. As shown there, the noise does not have any noticeable effect on the evolution of the non-diffracting wavefunction of the multi-electron beam, and the propagation dynamics is robust to deviations from non-ideal launch conditions.

Examining the diffraction of the multi-electron beam of Fig. 3e preserves its exact shape up to a propagation distance of *L*_d_ = 9.6 μm, and then the main lobe and the entire structure fade away quickly, within a short distance. Clearly, the non-diffraction range of the multi-electron non-diffracting beam of Figs. 1a and 3e is five times larger than the non-diffraction range of the multi-electron Bessel beam of Fig. 3d. Actually, the non-diffraction range of our non-diffracting multi-electron beam of Fig. 3e is closer to the corresponding range of the single-electron Bessel beam of Fig. 3b.

Altogether, as highlighted by Fig. 3, the wavefunction we find by seeking propagation-invariant solutions to the multiple-electron Schrödinger equation is indeed non-diffracting. It overcomes the repulsion among electrons and the natural tendency of diffraction broadening inherent in the Schrödinger equation. Moreover, when this wavefunction is launched with a finite BSS, it preserves its shape for a distance close to the range of the corresponding single-electron Bessel beam launched with the same BSS, in spite of the fact that the multi-electron beam carries very high current—corresponding to 74,000 electrons per cm. This fact implies that our non-diffracting multi-electron beams can be launched from the very same BSS and under the same noise conditions as Bessel EBeams are launched today in electron microscopes and in other applications.

### Non-diffracting range

Figure 4 presents a quantitative comparison in the performance between our non-diffracting multi-electron wavefunction and multi-electron Bessel and Gaussian beams, all carrying zero OAM. Figure 4 shows the non-diffraction range as a function of the effective width of the initial beam, with acceleration voltage of 200 V and current of 5 μA. The non-diffracting multi-electron beam (solid blue curve) performs remarkably better than the Gaussian beam (dotted blue curve) and also considerably better than the Bessel beam (dashed blue curve). The red curves display the current carried by the main lobe, solid for our non-diffracting wavefunction and dashed for the multi-electron Bessel beam. As shown there, the main lobe of the non-diffracting wavefunction carries more current than the Bessel beam, in addition to its better performance in terms of the non-diffraction range.

Interestingly, the dashed blue curve in Fig. 4 (describing the non-diffraction range of the Bessel beam) has a turn at 4.2 nm. We refer to this turn as the critical width (black dot-dashed vertical line), below which the performance of our non-diffracting wavefunction coincides with that of multi-electron Bessel beam (launched with the same BSS). We find that this critical width decreases as we increase the electron density in the beam (increasing the current), and it can go below 1 nm. This is because, in the region of very narrow multi-electron beams, that corresponds to *k*_*T*_ much larger than the inverse of the critical width, the potential satisfying Eq. (7) with initial condition $U\left( {\rho = 0} \right) = - k_T^2$ goes to a constant. This makes the wavefunction obeying Eq. (6) coincide with the Bessel function, hence—in this specific regime—their performances (non-diffraction range and current carried by the main lobe) coincide as well. However, in the entire other regime (where the width of the main lobe is larger than the critical width), the structure of our non-diffracting multi-electron beam is different than the Bessel wavefunction, and our non-diffracting beam performs much better than the Bessel beam, as highlighted by Fig. 4. The Supplementary Note 5 presents a comprehensive numerical study of the propagation of the beams as a function of the BSS.

Another important feature that can be seen in Fig. 4 is that the width of our non-diffracting wavefunction is bounded from above, at the blue dot (henceforth referred to as the maximal width), where the main lobe is over-wide. This over-wide wavefunction also marks the upper limit on the current. The reason for the existence of this upper limit is that the interference effects caused by the shape of our multi-electron wavepacket can balance the beam’s self-repulsion and diffraction only up to a certain electron density, above which the repulsion is too strong to be compensated by the predesigned interference effects. This upper limit point occurs for *k*_*T*_ = 0, which corresponds to the beam with the widest main lobe (Fig. 1b). The main lobe of this maximum-width non-diffracting wavefunction carries considerably higher current than the main lobe of the corresponding multi-electron Bessel beam.

**Fig. 5:**
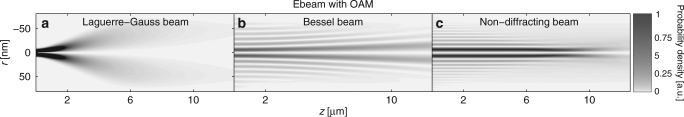
Evolution of multi-electron beams with non-zero OAM. **a**–**c** Propagation of a multi-electron beam with OAM = 1 and initial Laguerre–Gauss and Bessel wavefunctions, respectively. The repulsion among the electrons makes the Laguerre–Gauss EBeam diffracts very fast, while even the Bessel beam broadens considerably. **c** Propagation of the non-diffracting multi-electron beams with OAM = 1 and the wavefunction represented by the green curve in Fig. 1c. The beam preserves its shape for a large distance in spite of the repulsion among electrons

It is now interesting to study the propagation evolution of the shape-invariant multi-electrons beams that do carry OAM, such as those shown in Fig. 1c. Figure 5a–c present the simulated propagation of such wavefunctions (while neglecting the spin–spin and spin–orbit interaction; see Supplementary Note 3), with acceleration voltage of 200 V and current of 5 μA. Figure 5a, b show the propagation of initial wavefunction of Gaussian and Bessel shapes, with OAM = 1, respectively. Figure 5c presents the propagation of our non-diffracting wavefunction with OAM = 1. Similar to the case without OAM, the non-diffraction range of our non-diffracting beam (Fig. 5c) is much larger than the non-diffraction range of the Bessel beam (Fig. 5b). The performance of these multi-electron beams carrying OAM is similar to the trend shown in Fig. 4: the non-diffraction range is order of magnitude larger than for multi-electron Gaussian and Bessel beams, and is in fact similar to the non-diffraction range of the respective single-electron Bessel beam launched with the same BSS.

### Non-diffracting ultrashort-pulse EBeams

Finally, up to this point—the discussion was with continuous wave EBeams. However, one of the most important applications of high-density EBeams is in the ultrafast regime, when they are excited by an ultrafast optical pulse^24, 25, 28^. It is therefore important to study the 3D evolution of an ultrashort multi-electron pulse, which has the spatial shape of our non-diffracting wavefunction. A pulsed wavefunction has inherently a broad energy spectrum—because it is pulsed, therefore simulating the evolution of an ultrashort multi-electron pulse also examines the robustness to energy broadening and to modifications in the beam current (see further discussion in Supplementary Note 4 on the stability to modifications in the current and energy broadening).

**Fig. 6:**
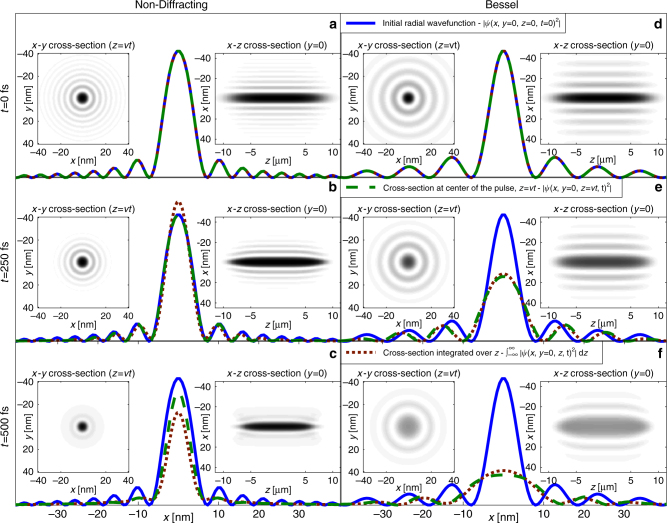
Temporal evolution of multi-electron pulsed beams. Shown are three frames taken from full 3D + time simulations. **a**–**c** present the 3D evolution of our non-diffracting wavefunction, while **d**–**f** show the 3D evolution of a Bessel beam. The *solid blue lines* show radial cross-sections of the beam at the launch plane *z* = 0 at *t* = 0, while the *green dashed lines* show beam cross-sections at the center of the pulse as the beam is evolving with *z* = *vt*. The red dotted lines show beam cross-sections integrated over the longitudinal (*z*) extent of the pulse, at different times, which corresponds to the quantity actually measured in experiments. Inserts display transverse shape (*x*-*y* cross-sections) of the wavefunctions (|*ψ*(*x*, *y*, *z* = = *vt*)|^2^) and longitudinal shape (*x*–*z* cross-sections) of the pulse (|*ψ*(*x*, *y* = 0, *z* − *vt*)|^2^). Our non-diffracting beam preserved its shape in space and time up to ~500 fs, while the Bessel beam expands considerably already at 200 fs

To this end, we simulate the 3D + time propagation of our non-diffracting multi-electrons beam and compare to that of a Bessel beam. Figure 6 shows the temporal evolution of such multi-electron pulsed beams. We design the 3D non-diffracting wavefunction as the solution of Eqs (6) and (7) (or the Bessel wavefunction) in the *x–y* plane and superimpose on it a super-Gaussian in the propagation direction *z*, as described by

13 $${\psi _{{\rm{non - diffracting}}}}\left( {{\bf{r}},t = 0} \right) = \frac{1}{{{a_0}}}\phi \left( \rho \right)\frac{{{e^{ - \frac{{{z^4}}}{{2{L^4}}}}}}}{{\sqrt L }},$$

14 $${\psi _{{\rm{Bessel}}}}\left( {{\bf{r}},t = 0} \right) = \frac{1}{{{a_0}}}{J_0}\left( {{k_T}\rho } \right)\frac{{{e^{ - \frac{{{z^4}}}{{2{L^4}}}}}}}{{\sqrt L }},$$

where *ϕ*(*ρ*) is the non-diffracting solution of eqs (6) and (7), *J*_0_ is the zero-order Bessel function, and *L* is the spatial extent of the pulse (pulse duration times group velocity).

The three columns in Fig. 6 show the evolution of the 3D wavefunctions at different times, *t* = 0 s, *t* = 250 fs and *t* = 500 fs. The left column in Fig. 6 presents the 3D evolution of our non-diffracting wavefunction, while the right column shows the 3D evolution of a Bessel beam. The simulations parameters are acceleration voltage of 20 kV, 100 electrons in a pulse of duration of ~200 fs (*L* = 100 fs), which is just about the shortest ultrashort pulses in electron microscopy^52–54^. Technically, launching such a non-diffracting ultrashort electron pulse would require incorporating the holographic scheme of Fig. 2 in the system of refs ^52–54^. The solid blue lines show radial cross-sections of the beam at the launch plane *z* = 0 at *t* = 0, while the green dashed lines show beam cross-sections at the center of the pulse, as the beam is evolving with *z* = *vt*. The red dotted lines show beam cross-sections integrated over the longitudinal (*z*) extent of the pulse, at different times, which corresponds to the quantity actually measured in experiments. Left inserts: transverse shape of the wavefunctions (|*ψ*(*x*, *y*, *z* = *vt*)|^2^). Right inserts: longitudinal shape of the pulse (|*ψ*(*x*, *y* = 0, *z* − *vt*)|^2^). Our non-diffracting beam preserves its shape in space and time up to ~500 fs, while the Bessel beam expands considerably already at 200 fs. It is worth noting that, as a result of the finite extent of the wavefunction in the *z* direction, the beam gets compressed at the beginning and at the end of the pulse (*z* = ±*L* from Eqs (13) and (14)), while in the central region it maintains its shape. The reason for the compression at the pulse edges is that the electron density there is low; hence, the repulsion among the electrons in these regions is weaker than at the pulse center, and the interference effects of the side lobes overcompensate the repulsion.

The Supplementary Movie 1 (Temporal evolution of multi-electron pulsed beams) displays a simulation, showing the 3D + time evolution, and Supplementary Movie 2 (Spectral evolution of multi-electron pulsed beams) depicting the evolution of the power-spectrum of the 3D wavefunctions, caused by the electron–electron interaction. The spectrum of the pulse is broadened during evolution—due to the nonlinear effects associated with the repulsion. More specifically, linear dispersion just affects the phases of the frequency components, but here we have a nonlinear interaction term (the repulsion between electrons) which does broaden the spectrum. It is important to note that the effect of this spectral broadening on the transverse shape of the non-diffracting multi-electron pulse is very minor (as shown in Fig. 6), because the shape of this EBeam is robust to spectral broadening (see Supplementary Movie 2).

## Discussion

Before closing, it is important to discuss the potential applications of our findings. Clearly, the non-diffracting multi-electron beams found here have inherent fundamental importance—similar to the impact made by the optical Bessel beam (which was the first non-diffracting beam discovered). In addition, the concept of non-diffracting multi-electron beams also has profound potential for applications, especially in electron microscopy. Specifically, the current in electron microscopes is proportional to the density of electrons. Converting to the spatial density only requires dividing by the velocity; hence, the nonlinear term in Eqs (6) and (7) is proportional to the current divided by the square root of the acceleration voltage (for nonrelativistic EBeams). Therefore, significant repulsion among electrons can arise either from high-current EBeam or from low-acceleration voltage. SEM and STEM work by focusing the EBeam on the sample under study, hence the resolution in both of them is determined by the diameter of focused spot (in TEM, the EBeam incident on the sample is broad, and the repulsion in that plane is negligible). Naturally, employing SEM and STEM in the high-current regime (tens to hundreds of micro-Amperes) would make the EBeam broaden after very short distances due to the repulsion^55^, which is exactly what our technique can counteract. Under realistic parameters of current SEM technology, our technique can increase the current density by at least factor 10^6^ while maintaining resolution of 1 nm (assuming that the high current does not damage the SEM components and the sample). In an alternative application, our method can be exploited in LEEM^56, 57^ to counteract the repulsive loss of resolution (that is especially significant due to the very low velocities). Likewise, our technique can be very important to microscopes working with ultrashort pulses of electrons (ultrafast electron microscopy)^24, 28^, where the electron density could be very high due to the ultrashort duration of the pulse.

When working with ultrafast pulses of multi-electron beams, it is important to note that, even though our non-diffracting beam is designed for a monochromatic source, the 3D simulation shows that the actual pulse—which has a considerable bandwidth—works well as a non-diffracting beam. For the same reason, chromatic aberrations, which often occur in microscopes with high electron densities, do not pose a problem: the beam is non-diffracting even if its bandwidth is considerably expanded by the chromatic aberations and space charge field effects in the propagation direction.

To conclude, we have shown that the wavefunctions of multi-electron beams, or any other beams of charged particles, e.g., protons, muons and ion beams, can be properly designed to compensate for both space-charge (self-repulsion) effects and diffraction broadening, and can even carry OAM. Our simulations predict that our shaped non-diffracting beams perform remarkably better than the multi-electron Bessel and Gaussian EBeams. The design methodology presented here finds applications in electron microscopy, electron beam lithography, accelerators and a variety of other applications. Using our shaped multi-electron beams in low-energy and high-current microscopes, one can still achieve high resolution despite the repulsion among the electrons. Essentially, what we suggest here can resolve the space-charge field effects that appear in all technologies using beams of multiple electrons. In this vein, we also present a full-scale simulation of the 3D+time evolution of an ultrashort electron pulse, which has inherently a broad energy spectrum. Still, our wavefunction preserves its shape despite the broad energy spectrum. Finally, we recall the resemblance of our model for multi-electron beams to the Newton–Schrödinger model known from General Relativity (with the exception that the force in our EBeam is repulsive, whereas the force in the Newton–Schrödinger model is attractive). We also note the similarity of our non-diffracting multi-electron beams to solitons in nonlocal nonlinear media in optics and in cold atomic dipoles. These resemblances raise a series of intriguing questions, among them: the existence of dark solitons made of multi-electron beams, and long-range interactions among such self-trapped entities.

### Data availability

The data that support the findings of this study are available from the corresponding author upon reasonable request.

## Electronic supplementary material

Supplementary Information

Supplementary Movie 1

Supplementary Movie 2
