# Supplementary material for: Non-diffracting multi-﻿electron vortex beams balancing their electron–electron interactions
Source: Nat Commun. 2017 Sep 21;8:650. doi: 10.1038/s41467-017-00651-z (PMC5608825; doi:10.1038/s41467-017-00651-z)
Supplement: Supplementary file 1 — Supplementary Information [file 41467_2017_651_MOESM1_ESM.pdf]

Description of Supplementary Files

File Name: Supplementary Information
Description: Supplementary Notes, Supplementary Figures, Supplementary References

File Name: Supplementary Movie 1
Description: This movie displays the temporal evolution of multi-electron pulsed beams.

File Name: Supplementary Movie 2
Description: This movie displays the spectral evolution of multi-electron pulsed beams.

Supplementary Note 1: Derivation of the equation of the non-diffracting beam

This section described the derivation of the equation governing the propagation of the multi-electron beam.

The exact Schrödinger equation describing the evolution of a beam of multiple electrons contains a nonlinear set of coupled equations, whose number is the same as the number of electrons. To make the problem tractable, we approximate the full multi-electrons Hamiltonian by the Hartree Hamiltonian, which is an effective mean-field Hamiltonian. This approach assumes that the influence of the fermionic nature of the electrons (the exclusion principle) is very weak. For example, this issue is studied in [1], which used an EBeam with very high electron density (similar to the densities studied here), and confirmed that indeed that the exclusion principle plays only a minor role under these conditions. As stated there: The particle beam has low degeneracy (about 10^{-4} electrons per cell in phase space)… (Therefore) To prove antibunching, a total of four spectra were accumulated for about 30h each. This means that exclusion principle plays only a minor role. Likewise, it was shown [2] that, although the fermionic effect on the quantum evolution of a pulsed EBeam can be observed in experiments, it is largely insignificant. Hence, we are allowed to take the simplest case where all the electrons have the same wavefunction, as happens naturally in electron microscopes.

The Schrödinger Equation for a system of multiple electrons under the Hartree (mean-field) Hamiltonian, is as follows:

$$-i\hbar\partial_t\psi_i(\mathbf{r},t) = -\frac{\hbar^2}{2m}\nabla^2\psi_i(\mathbf{r},t) + \frac{e^2}{4\pi\varepsilon_0}\sum_{j=1,j\neq i}^{N}\left(\int \frac{|\psi_j(\mathbf{r}',t)|^2}{|\mathbf{r}-\mathbf{r}'|}d^3\mathbf{r}'\right)\psi_i(\mathbf{r},t). \quad (1)$$

Where, ψ_i and is the wavefunction of the ith electron, $\hbar$ is the reduced Planck constant, m and e are the mass and charge of the electron respectively, ε_0 is the vacuum permeability and N is the total number of electrons in the EBeam.

We note that relativistic electrons (such as in TEM) can still be described by the Schrödinger Equation, with a modified mass (see the appendix in [3]). Moreover, a multi-electron beam, as any beam of charged particles, has an additional self-focusing effect by the magnetic field the beam itself creates. Here, we calculate the wavefunctions in the rest frame of the electrons, where this effect is zero. In the lab

frame this magnetic field effect can be seen as length contraction. However, this effect is very small in the case analyzed here, due to low electron velocities.

Next, we assume that all the electrons have the same wave function, as reflected by Eq. 1 in the main text. We also assume that the self-interaction (when *j*=*i*) is negligible, which is the case when the beam is very dense (N is a large number). Proceeding to substitute the wavefunction from Eq. 1 and the potential form from the main text, we recover Eqs. 6, 7 there, which in cylindrical coordinates are:

$$-\left(\frac{1}{\rho}\partial_\rho\left(\rho\partial_\rho\right)-\frac{l^2}{\rho^2}\right)\phi(\rho)+\frac{1}{a_0^2}U(\rho)\phi(\rho)=\left(\frac{2m}{\hbar^2}E-k^2\right)\phi(\rho) \tag{2}$$

$$\frac{1}{\rho}\partial_\rho\left(\rho\partial_\rho\right)U(\rho)=-\frac{8\pi n}{a_0}|\phi(\rho)|^2 \quad . \tag{3}$$

It is now convenient to define $U(0)=a_0^2\left(\frac{2m}{\hbar^2}E-k^2\right)$, which simplifies Supplementary Eq. (2) to Eq. 4 from the main text:

$$\left(\frac{1}{\rho}\partial_\rho\left(\rho\partial_\rho\right)-\frac{l^2}{\rho^2}\right)\phi(\rho)=\frac{1}{a_0^2}U(\rho)\phi(\rho) \tag{4}$$

The normalization requirement is:

$$2\pi\int_{\mathrm{BSS}}|\phi(\rho)|^2\rho d\rho=1 \tag{5}$$

The initial conditions for the nonlinear set of equations are:

$$\begin{cases}\phi(0)=\alpha\\ \phi'(\varepsilon)=\alpha k_T J_l'(k_T\varepsilon)\\ U(0)=-k_T^2\\ U'(0)=0\end{cases} \tag{6}$$

where α is determined from the normalization requirement.

To find α that satisfies Supplementary Eq. (5), we use an iterative algorithm. In the first iteration, the algorithm guesses the value of α and finds the wavefunction using the 'ode45' matlab procedure (the algorithm is an explicit Runge-Kutta formula). From there on, in each iteration k, we update the value of α_k to α_{k+1} as $\alpha_{k+1}=\alpha_k\left(1+sign\left(1-2\pi\int|\phi_k(\rho)|^2\rho d\rho\right)\left|1-2\pi\int|\phi_k(\rho)|^2\rho d\rho\right|^r\right)$, where we use $r=1.2$. The fact that $|\phi(\rho)|^2$ is symmetric in space implies that $U'(0)=0$, hence the only free parameter in Supplementary Eqs. (3) and (4) is k_T - which can vary between 0 and infinity. At the vicinity of $\rho=0$, Supplementary Eq. (4) gives the Bessel equation, whose solution is $\phi(\rho)=\alpha_1 J_l(k_T\rho)+\alpha_2 Y_l(k_T\rho)$. However, $Y_l(k_T\rho)$, is unphysical because it diverges at $\rho=0$. As such, we are left with the first term only, which is the reason why k_T is the only remaining degree of freedom.

The electron density on the z axis of an EBeam (as appears in Eq. 5 in the main text) can be derived from the current I, and the acceleration voltage V:

$$n = \frac{dN}{dz} = \frac{1}{e}\frac{dq}{dz} = \frac{1}{e}\frac{dq}{dt}\frac{dt}{dz} = \frac{I}{ev} = \frac{I}{\sqrt{V}}\sqrt{\frac{m}{2e^3}}. \tag{7}$$

As a side note, we would like to add that, while the above treatment is non-relativistic, it can be directly extended to a fully relativistic quantum formalism. In case the propagation is limited to small angles (paraxial EBeams), the Schrödinger Equation only needs to be changed by multiplying the mass by the relativistic gamma, and decreasing the interaction terms by the same factor. In any case, the non-relativistic equation above is a very good approximation for the parameters we simulate in the paper.

Supplementary Note 2: Code for simulating the evolution of spatio-temporal electron pulses and beams

2.1: Beam propagation code for spatio-temporal electron pulses

To simulate the evolution of the wavefunction in time and 3D space, which includes charge distribution in full 3D, we use a modified version of the commonly-used Beam Propagation Method (BPM). We begin with Eq. 4 from the main text.

$$\partial_t \psi(\mathbf{r},t) = i\frac{\hbar}{2m}\left(-\nabla^2 + \frac{1}{a_0^2}U(\mathbf{r},t)\right)\psi(\mathbf{r},t). \tag{8}$$

Then, we define the diffraction operator and the non-linear operator, as

$$\widehat{D} = -i\frac{\hbar}{2m}\nabla^2\,;\ \widehat{N} = i\frac{\hbar}{2m}\frac{1}{a_0^2}U_{3D}(\mathbf{r},t). \tag{9}$$

The equation of motion becomes:

$$\partial_t \psi(\mathbf{r},t) = \left(\widehat{D} + \widehat{N}\right)\psi(\mathbf{r},t). \tag{10}$$

For a small propagation step in time, *dt* , the solution of Eq. (10) is

$$\psi(\boldsymbol{r},t+dt) = e^{(\widehat{D}+\widehat{N})dt}\psi(\mathbf{r},t). \tag{11}$$

Note, that the diffraction operator $\widehat{D}$ is now diagonal in momentum space, while the non-linear operator $\widehat{N}$ is diagonal in 3D real space. Therefore, we cannot evolve both operators together as diagonal matrices. We therefore use the Baker-Campbell-Hausdorff theorem:

$$e^{\widehat{D}dt}e^{\widehat{N}dt} = e^{\widehat{D}dt+\widehat{N}dt+[\widehat{D},\widehat{N}]\frac{dt^2}{2}+O(dt^3)}, \tag{12}$$

where, for small enough *dt* we get $e^{(\widehat{D}+\widehat{N})dt} \approx e^{\widehat{D}dt}e^{\widehat{N}dt}$, hence

$$\psi(\mathbf{r},t+dt) \approx e^{\widehat{D}dt}e^{\widehat{N}dt}\psi(\mathbf{r},t) = e^{-idt\frac{\hbar}{2m}\nabla^2}e^{idt\frac{\hbar}{2m}\frac{1}{a_0^2}U(r,t)}\psi(\mathbf{r},t). \tag{13}$$

To evolve the diffraction operator $e^{-idt\frac{\hbar}{2m}\nabla^2}$, we transform the wavefunction to the momentum space, where this operator is of the form $e^{-idt\frac{\hbar}{2m}\boldsymbol{k}^2}$. The evolution step in a time element dt is:

$$\psi(\mathbf{r},t+dt) = \mathcal{F}_{3D}^{-1}\left\{e^{-idt\frac{\hbar}{2m}\mathbf{k}^2}\mathcal{F}_{3D}\left\{e^{idt\frac{\hbar}{2m}\frac{1}{a_0^2}U(\mathbf{r},t)}\psi(\mathbf{r},t)\right\}(\mathbf{k})\right\}. \quad (14)$$

where the potential U is calculated by solving numerically Eq. 5 from the main text:

$$\nabla^2 U(\mathbf{r},t) = -8\pi N a_0|\psi(\mathbf{r},t)|^2. \quad (15)$$

The calculation is performed in momentum space, as follows:

$$U(\mathbf{r},t) = \mathcal{F}_{3D}^{-1}\left\{\frac{1}{\mathbf{k}^2}\mathcal{F}_{3D}\{-8\pi N a_0|\psi(\mathbf{r},t)|^2\}(\mathbf{k})\right\}. \quad (16)$$

This procedure is used to simulate the evolution in time and 3D space of a pulsed electron beam whose charge distribution is in full 3D.

2.2: Beam propagation code for continuous wave electron beams

Similar to the evolution of the electron pulse in time and 3D space, we simulate the evolution of continuous wave (CW) electron beam in time and 2D space. Again, we use a modified version of BPM. We begin with Eq. 4 from the main text.

$$\partial_t\psi(\mathbf{r},t) = i\frac{\hbar}{2m}\left(-\nabla^2 + \frac{1}{a_0^2}U(\mathbf{r},t)\right)\psi(\mathbf{r},t). \quad (17)$$

Note, that here the wavefunction evolves in time in a non-harmonic fashion (i.e., it does not evolve with $e^{-i\omega t}$). Rather, the evolution in time depends on the initial condition. Next, we transform (17) to cylindrical coordinates

$$\psi(\mathbf{r},t) = \frac{1}{a_0}\Phi(\rho,\theta,t)\frac{e^{ikz}}{\sqrt{L}}. \quad (18)$$

The equation of motion obtains the following form:

$$\partial_t\Phi(\rho,\theta,t) = i\frac{\hbar}{2m}\left(-k^2 - \boldsymbol{\nabla}_\perp^2 + \frac{1}{a_0^2}U(\rho,t)\right)\Phi(\rho,\theta,t). \quad (19)$$

Where, $\boldsymbol{\nabla}_\perp^2$ is the Laplacian in the transverse plane. Then, we define the diffraction operator and the non-linear operator, as

$$\widehat{D} = -i\frac{\hbar}{2m}\boldsymbol{\nabla}_\perp^2\,;\ \widehat{N} = i\frac{\hbar}{2m}\frac{1}{a_0^2}U(\rho,t). \quad (20)$$

For a small propagation step in time, dt

$$\Phi(\rho,\theta,t+dt) \approx e^{-idt\frac{\hbar}{2m}\boldsymbol{\nabla}_\perp^2}e^{idt\frac{\hbar}{2m}\frac{1}{a_0^2}U(\rho,t)}\Phi(\rho,\theta,t). \quad (21)$$

Again the diffraction operator $e^{-idt\frac{\hbar}{2m}\nabla_\perp^2}$, in the momentum space, is of the form $e^{-idt\frac{\hbar}{2m}k_\perp^2}$, where $\mathbf{k}_\perp^2$ is the transverse wavenumber, squared. The evolution step in a time element dt is:

$$\Phi(\rho,\theta,t+dt)=\mathcal{F}_{2D}^{-1}\left\{e^{-idt\frac{\hbar}{2m}\mathbf{k}_\perp^2}\mathcal{F}_{2D}\left\{e^{idt\frac{\hbar}{2m}\frac{1}{a_0^2}U(\rho,t)}\Phi(\rho,\theta,t)\right\}(\mathbf{k}_\perp)\right\}. \tag{22}$$

where the potential U is calculated by solving numerically Eq. 5 from the main text:

$$\nabla_\perp^2 U(\rho,t)=-\frac{8\pi n}{a_0}|\Phi(\rho,\theta,t)|^2. \tag{23}$$

Where, n is the electron density on the z axis of an EBeam (as appears in Supplementary Eq. 7). The calculation is performed in momentum space, as follows:

$$U(\mathbf{r},t)=\mathcal{F}_{2D}^{-1}\left\{\frac{1}{\mathbf{k}_\perp^2}\mathcal{F}_{2D}\left\{-\frac{8\pi n}{a_0}|\Phi(\rho,\theta,t)|^2\right\}(\mathbf{k}_\perp)\right\}. \tag{24}$$

Supplementary Note 3: Neglecting the spin-spin and spin-orbit interaction

This approach is along the lines of previous work addressing related questions (see, reference [4]) that showed that, in most standard EBeam conditions, the coulomb interaction dominates over any spin-related effect. Here, the energy of the spin-spin and spin-orbit interaction is as follows:

$$E_{\text{spin}}\sim\frac{Ne^2h^2}{4\pi\varepsilon_0 m^2c^2}\langle\frac{1}{r^3}\rangle \tag{25}$$

While the energy related to the potential energy of Coulomb interaction is:

$$E_{e-e}\sim\frac{Ne^2}{4\pi\varepsilon_0}\langle\frac{1}{r}\rangle \tag{26}$$

The ratio between them is:

$$\frac{E_{e-e}}{E_{\text{spin}}}\sim\frac{m^2c^2}{h^2}\frac{\langle\frac{1}{r}\rangle}{\langle\frac{1}{r^3}\rangle}=\frac{L_{\text{typical}}^2}{\lambda_c^2} \tag{27}$$

Where, L_{typical} is a typical length scale in the system and λ_c is the Compton wavelength. The EBeams we consider have two typical lengths: BSS in the x,y plane, and the average distance between electrons $(1/n)$ along the z axis. Both are typically $1nm-1\mu m$ or even larger, while the Compton wavelength is $\lambda_c=2.4pm$. This means that the spin-spin and spin-orbit interactions are negligible in this system.

$$\frac{E_{e-e}}{E_{\text{spin}}}\sim\frac{L_{\text{typical}}^2}{\lambda_c^2}\gg 1. \tag{28}$$

In a similar vein, previous work that compared the spin-spin interaction and the coulomb repulsion led to similar conclusions (see ref. [4]): in most standard EBeam conditions the spin-spin interaction is negligible relative to the space charge effect.

Supplementary Note 4: Stability to modifications in the current and energy-broadening effects

In this section, we show in simulations that our non-diffracting wavefunction is stable under variations in the density in the EBeam. Namely, we show in simulations that our non-diffracting wavefunction is robust to modifications in the current and against energy broadening. In a physical setting, it would be expected that our non-diffracting wavefunction would be stable against energy broadening and would be able to adjust to modifications in the current.

In next figure, we actually examine the evolution of our non-diffracting wavefunction with a current different from the current it was designed for. We take the wavefunction that solves Supplementary Eqs. (3), (4) with a current of $5\mu A$, and acceleration voltage of $200V$ (where it exhibits non-diffracting propagation; upper panel below). After that we let it evolve with a beam density that relates to a beam current of $6\mu A$ and acceleration voltage of 200V (middle panel below). This simulation also shows robustness to energy variation. In Supplementary Eq. (7) we show that the beam density is proportional to the current divided by the square root of the acceleration voltage. Therefore, in the same simulation we show also the evolution of a beam with current of 5μA and energy of 140eV. We also demonstrate the evolution of a beam with a current of $7.5\mu A$ and acceleration voltage of 200V (lower panel below). One can see that the beam in the lower panel is non-diffracting up to distance comparable to the beam in the upper panel. This robustness takes place up to 50% variations in the current or 60% variations in the acceleration voltage.

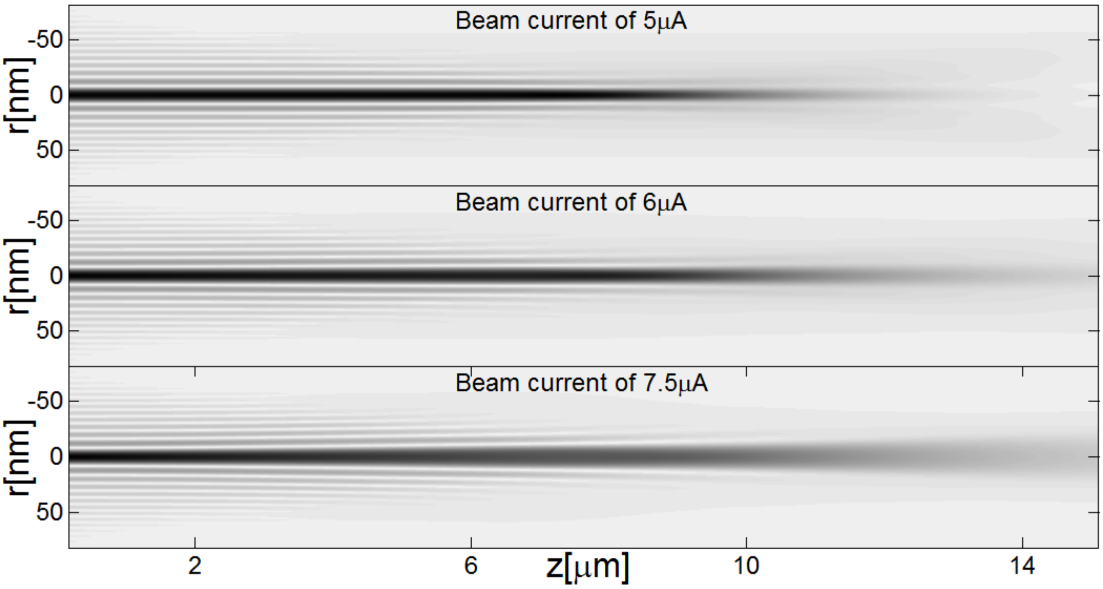

Supplementary Figure 1: Stability of the non-diffracting wavefunction to modifications in electron density. All panels show the evolution of non-diffracting wavefunction that solves Supplementary Eqs. (3), (4) with a current of $5\mu A$, and acceleration voltage of $200V$. The upper panel shows the evolution of an EBeam with a current of $5\mu A$, and acceleration voltage of $200V$. The middle panel shows the evolution of an EBeam current of $6\mu A$ and acceleration voltage of 200V. The lower panel shows the evolution of an EBeam current of $7.5\mu A$ and acceleration voltage of 200V. In all three cases, the EBeam displays non-diffracting properties for similar distances, even though the wavefunction is designed for the specific case of the upper panel (current of $5\mu A$ and acceleration voltage of $200V$).

Supplementary Note 5: Non-diffracting range and effective current vs. beam width, for different BSS

The following figure presents a quantitative comparison in the performance between our shape-preserving multi-electron wavefunction and multi-electron Bessel and Gaussian beams. Similar to the compassion that was made in Fig. 4 in the paper but with lower beam current of $500nA$ (recall the total beam current in Fig. 4 in the paper is $I = 5\mu A$). We present the results for two BSS: $140nm$ (as in the example in Fig.3) and $420nm$, both under the same acceleration voltage of $200V$. The BSS determines the wavefunction density because of normalization requirement from Eq. (5). Therefore, the BSS size affects the nonlinear solution and the non-diffracting range of the EBeam, even for a fixed beam current.

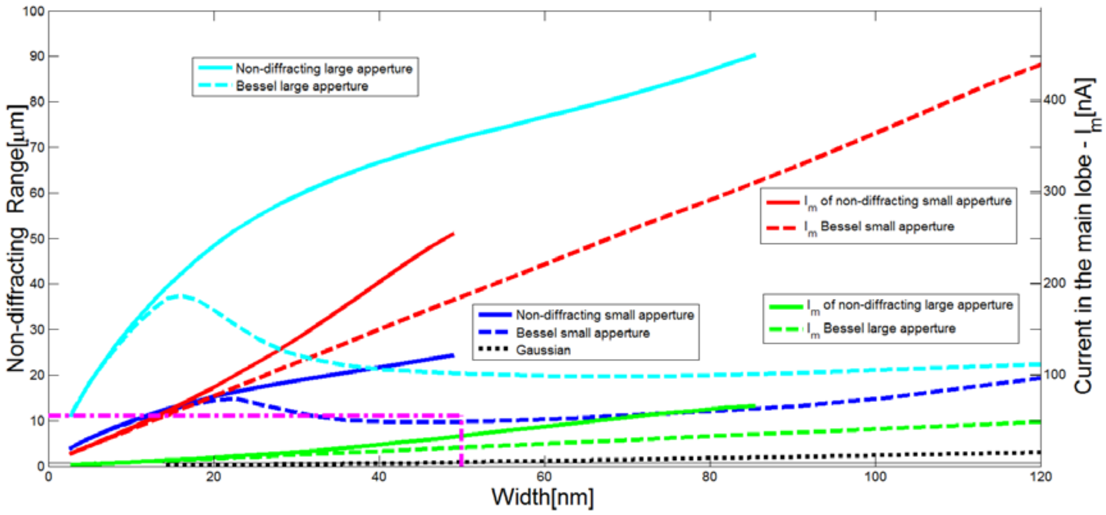

Supplementary figure 2: Non-diffraction range and effective current vs. beam width for different BSS. The wavefunctions are designed for BSS of $140nm$ and $420nm$ and total current of $500nA$. The figure displays the non-diffracting range for the three types of initial wavefunctions, with the shape-preserving wavefunction in solid lines, the Bessel beam in dashed lines, and the Gaussian beam in black dotted line. The figure compares the case of wide BSS (cyan curves) and small BSS (blue curves). The current inside the main lobe of the beam is denoted in green for the large BSS and in red for the small BSS.

We can see, in Supplementary figure 2, tendencies similar to the results presented in Fig. 4. However, the Critical width here is much larger, showing strong dependence on the BSS, while only weak dependence on the total current. Additionally, as the BSS is increased, the EBeam density decreases (because of total probability normalization) and the EBeam experiences less repulsion, resulting in larger non-diffracting range. This figure explains the larger non-diffracting range of the wide BSS when compare to the smaller BSS (see cyan vs. blue curves), but also leads to the lower current inside the main lobe (green vs. red). Further comparison is shown in Fig. 4 in the main text, which is the zoom-in of the area marked by the magenta rectangle.

Supplementary References

1. Kiesel, H., Renz, A. & Hasselbach, F. Observation of Hanbury Brown–Twiss anticorrelations for free electrons. *Nature* **418,** 392–394 (2002).

2. Lougovski, P. & Batelaan, H. Quantum description and properties of electrons emitted from pulsed nanotip electron sources. *Phys. Rev. A* **84,** 23417 (2011).

3. Voloch-Bloch, N., Lereah, Y., Lilach, Y., Gover, A. & Arie, A. Generation of electron Airy beams. *Nature* **494,** 331–5 (2013).

4. Lobastov, V. A., Srinivasan, R. & Zewail, A. H. Four-dimensional ultrafast electron microscopy. *Proc. Natl. Acad. Sci. U. S. A.* **102,** 7069–73 (2005).
